# Distribution of human papilloma virus genotype prevalence in invasive cervical carcinomas and precancerous lesions in the Yangtze River Delta area, China

**DOI:** 10.1186/s12885-018-4330-9

**Published:** 2018-04-27

**Authors:** Hongyun WANG, Xiaodong CHENG, Jing YE, Xiuyun XU, Ying HONG, Long SUI, Zhixue YOU, Xing XIE

**Affiliations:** 1 Department of Gynecological Oncology, Women’s Hospital, School of Medicine, Zhejiang University, Zhejiang, China; 2 Nanjing Drum Tower Hospital, the Affiliated Hospital of Nanjing University Medical School, Nanjing, China; 3 Obstetrics and Gynecology Hospital of Fudan University, Fudan, China; 4 The First Affiliated Hospital of Nanjing Medical University, Nanjing, China

**Keywords:** Cervical intraepithelial neoplasia (CIN), Human papilloma virus (HPV), Invasive cervical cancer (ICC)

## Abstract

**Background:**

This study aimed to provide more information for cancer prevention strategies by determining the distribution of human papilloma virus (HPV) genotype prevalence in invasive cervical carcinoma (ICC) and precancerous lesion patients in the Yangtze River Delta area in China.

**Methods:**

This multi-centre descriptive cross-sectional study involves four university hospitals in the Jiangzhehu area. Women with histologically confirmed cervical intraepithelial neoplasia (CIN) 1, CIN2, CIN3 or ICC who were diagnosed and treated in the four selected hospitals between February 2012 and April 2014 were eligible for recruitment. The average age of the patients was 40.93 ± 11.87 years old, among whom the youngest was 17 years old and the oldest was 76 years old.Those with immunodeficiency diseases or a previous history of cancer or CIN were excluded. HPV genotyping was performed by a central laboratory. The distribution and age and disease specificity of the HPV genotype prevalence were analysed.

**Results:**

Of the 2181 collected samples, 251 were ICC and 1930 were CIN. The mean age of cervical cancer and CIN patients was 40.93 ± 11.8 years (range, 17–76 years). The five most commonly identified HPV types in each lesion class were as follows: CIN1: 52, 58, 16, 33, and CP; CIN2: 16, 58, 52, 33, and 31; CIN3: 16, 58, 33, 52, and 31; and ICC: 16, 58, 18, 52, and 33. CIN1 had an earlier age of onset (30–40 years) than CIN2, CIN3, and cervical cancer. The age of onset of cervical cancer exhibited two peaks at 40–44 and 50–54 years of age. In all infected patients, the frequency of HPV infection with a single type was 62.9%, and with multiple types, it was 38.1%. There was no difference in the frequencies of multiple types amongst the different cervical lesions.

**Conclusions:**

The most prevalent genotypes in the investigated area (52, 58, 16 and 18) justify the necessity of anti-HPV vaccination in teenagers and young girls under 24 years old in the Yangtze River Delta area in China. Infection with multiple high-risk HPV types versus single infection does not increase the risk for ≥ CIN2 in ICC development.

## Background

Cervical cancer is the third most common cancer amongst women worldwide, with an estimated 500,000 new cases annually and a 50% mortality rate[1]. Human papillomavirus (HPV), the most common sexually transmitted virus [2], has been identified as a major risk factor for both ICC and cervical intraepithelial neoplasia (CIN). CIN1 has the risk of developing into ≥ CIN2, and ≥ CIN2 has the risk of developing into ICC; ≥ CIN2 is also known as precancerous lesions. A recently developed HPV vaccine is effective in the prevention of cervical neoplasia. One quadrivalent and one bivalent vaccine targeting genital HPV have been effectively applied for the prevention of CIN in over 100 countries [3, 4]. Additionally, a 9-valent vaccine is currently available. However, the effect of prophylactic vaccines is type-specific. Both currently licensed vaccines prevent infection of the most prevalent HPV types (types 16, 18, 6, and 11), but they do not cover all high-risk HPV (HR-HPV) types associated with cervical cancer. Furthermore, the effective prevention of HPV types covered by a vaccine may affect their distribution. Therefore, determining the distribution of HPV types before beginning a vaccination programme will provide baseline information enabling the evaluation of the effectiveness of mass vaccination and the identification of a change in type distribution. Because it is impossible to investigate all HPV types, clarification of the most carcinogenic types of HPV will provide important knowledge for the development of a new generation of HPV vaccines.

Although infection with HPV is common while ICC is rare, persistent infection with high-risk or oncogenic types increases the risk of developing cancer. International prevalence studies estimate that approximately 70% of ICCs are attributed to either HPV-16 or − 18 [5–8]. A meta-analysis of 85 studies worldwide revealed that the overall rate of HPV infection in ICC patients was similar in all regions (83–89%). HPV-16 was the predominant type in squamous cell carcinoma (SCC; 46–63%) followed by HPV-18 (10–14%). The predominant type in adenocarcinoma (ACa) and adenosquamous carcinoma (AdCa) was HPV-18 (37–41%) followed by HPV-16 (26–36%) [6]. HPV-16/18 accounted for 52% of infections in high-grade cervical lesions [9]. However, the type distribution of HPV infection varies widely across the world [2, 9, 10]. Elucidation of the type distribution of HPV will aid the development of public health policy programmes and provide evidence for the future selection of vaccines targeting HPV types common to a specific region. Despite these obvious benefits, data concerning HPV distribution are rarely reported in most underdeveloped countries or regions.

The Yangtze River Delta region (Jiangzhehu area) includes Jiangsu Province, Zhejiang Province, and Shanghai; it is the most economically developed region in China. The total GDP in the Yangtze River Delta area was approximately $1 trillion USD in 2010 [11], and the per capita GDP has exceeded $4000 USD since 2005 [12]. This provides strong economic support for the widespread use of the HPV vaccination. Additionally, the population possesses a high awareness of health issues and has the highest demand for self-expense vaccination in China. Considering the shortage of medical care in China, the HPV vaccine has not been widely used. However, it can be used in the economically developed Yangtze River Delta Region because of self-funded vaccination. Because clinical trials of both HPV vaccines are nearing completion in China, elucidating the type-specific distribution of HR-HPV in the region prior to the implementation of mass vaccination programmes is prudent and will provide scientific evidence for the future development of a new generation of vaccines suitable for this region.

In this cross-sectional study, cervical specimens from ICC and CIN patients residing in the Delta region were collected. HPV genotypes were determined, and the type distribution was analysed amongst the population. This study aimed to understand the distribution of HPV types and to provide comprehensive scientific evidence to aid the development of control strategies and prevent cervical cancer in the region.

## Methods

### Material

Suitable patients who visited one of four university-affiliated hospitals within the region were enrolled in this study. These hospitals included the Women’s Hospital, Zhejiang University School of Medicine, the Affiliated Drum Tower Hospital of Nanjing University School of Medicine, the Obstetrics and Gynaecology Hospital of Shanghai Fudan University, and the First Affiliated Hospital of Nanjing Medical University. The cases came from 4 centres of the Yangtze River Delta area in China. These four central hospitals represent the top hospitals of Zhejiang, Jiangsu and Shanghai. According to the number of patients with local illnesses, more than half of the local women in this area undergo abnormal and opportunistic screening for cervical cancer in the clinics of these four hospitals. Approximately 15% of the local population participated in cervical cancer screening. All hospitals have sufficient patient resources to reflect the HPV classification of cervical cancer and precancerous lesions and suitable clinical and laboratory facilities to undertake this study.

The study population comprised patients diagnosed with CIN1, CIN2, CIN3, or ICC based on pathology who were treated at one of the four aforementioned hospitals between February 2012 and April 2014. To be included in the study, patients were required to meet the following inclusion criteria: untreated histopathological diagnosis of CIN1, CIN2, CIN3, or ICC; no previous ICC or CIN history; negative for immunodeficiency diseases, including HIV; informed consent; and permanent residency in the Yangtze River Delta region. The average age of the patients was 40.93 ± 11.87 years old, among whom the youngest was 17 years old and the oldest was 76 years old. There were no patients younger than 16 years old. There were 10 excluded patients whose postoperative diagnoseis didn’t accord with preoperative diagnoses (3 CIN1 cases,2 CIN2 cases, 3 CIN3 cases,and 2 ICC cases).

Pathological diagnosis of cervical lesions was used as the gold standard, and the diagnosis was conducted and reviewed in each hospital independently. Uniform diagnostic review was not conducted. Diagnosis of cervical cytology was classified by the 2001 revised Bethesda system. Pathology experts interpreted the results as follows: absence of intraepithelial lesion or malignancy (WLM); atypical squamous cells of undetermined significance (ASCUS or AGUS); atypical squamous cells, high-grade intraepithelial lesions cannot be excluded (ASC-H); low-grade squamous intraepithelial lesion (LSIL); high-grade squamous intraepithelial lesion (HSIL), including those caused by HPV infection; and SCC, ACa, and AdCa. Histological grading was based on Broder’s classification.

### Methods research protocols

A cross-sectional study design was employed to determine the distribution of HPV genotypes amongst ICC and CIN patients in the Delta region. According to the histopathological results, patients were divided into 4 groups: the CIN1, CIN2, CIN3 and ICC groups. The patient information was collected and then tested for HPV typing.

### Specimen collection

All participating hospitals followed the same patient registration protocol and obtained the following information: patient name, assigned patient number, completed HPV patient information collection form (CRF), and signed informed consent. The survey was approved by the Medical Ethics Committee of Affiliated Obstetrics and Gynecology Hospital of Zhejiang University Medical School(or called Women’s Hospital School Of Medicine Zhejiang University). Cervical cytology was conducted for all patients following a gynaecological examination. The study was conducted over a period of 26 months. For consistency of results and to ensure proficiency, all registrations were completed by a physician and a trained investigator at each hospital.

All participating hospitals sequentially registered patients, and physicians recorded all information in the HPV-CRF. According to the histopathological results, patients were divided into 4 groups: the CIN1, CIN2, CIN3 and ICC groups. The cervical exfoliated cells collected before operation were genotyped for HPV, 10 cases were excluded because of the inconformity of the pre- and post-operative diagnoses. A Cytobrush was inserted into the endocervical canal until the brush bent against the ectocervix. The brush was then rotated clockwise five times. After specimen collection, the brush head was placed into a collection vial that was labelled with the collection date, patient name, and patient number before the HPV types was checked. The storage vial with the brush head was stored immediately at − 20°C. All specimens were sent to a central laboratory within weeks of collection for determination of the HPV genotype.

### Determination of HPV genotype

HPV genotyping was conducted as reported previously [13].

### Real time-PCR for HR-HPV DNA

An HPV GenoArray test was performed for the HR-HPV samples. The HPV genotype was determined by DNA amplification, flow-through hybridization, and a gene chip using the 21 HPV GenoArray Diagnostic Kit (HybriMax, Chaozhou Hybribio Limited Corp., Chaozhou, China) according to the manufacturer’s instructions. Detailed protocols for this assay have been described previously [13]. The gene chip contained type-specific oligonucleotides immobilized on a nylon membrane. The chip could identify 13 HR-HPVs (16, 18, 31, 33, 35, 39, 45, 51, 52, 56, 58, 59, and 68), five low-risk HPVs (LR-HPVs) (6, 11, 42, 43, and 44), and other HPV types found in Chinese populations (53, 66, and 81/CP8304). The results were detected by colorimetric changes on the chip under direct visualization.

All results were verified using the 37 HPV GenoArray Diagnostic Kit from the same company. The chip could identify 15 HR-HPVs (16, 18, 31, 33, 35, 39, 45, 51, 52, 53, 56, 58, 59, 66, and 68) and 24 LR-HPVs (6, 11, 26, 34, 40, 42, 43, 44, 54, 55, 57, 61, 67, 69, 70, 71, 72, 73, 82, 83, 84, and CP8304).

### Statistical analysis

A database was established using Excel 2003, and the results were analysed using SPSS 17.0 software (SPSS Inc., Chicago, IL, USA). A Chi-square test was used for the counting analysis, and a *t*-test was used for variable data. A *p* value of < 0.05 was considered significant.

## Results

### Distribution of HPV types in CIN and ICC women from the Yangtze River Delta region

**Fig. 1:**
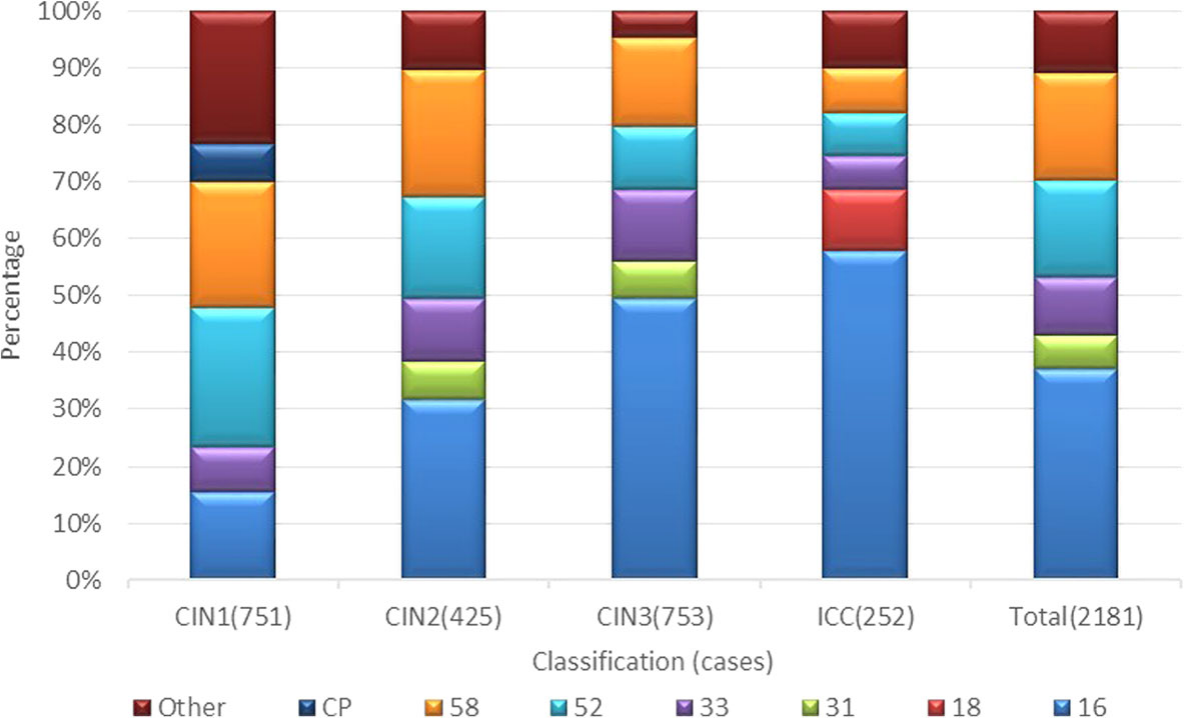
Distribution of HPV infection with a single type and multiple types in women from the Yangtze River Delta region. (HPV)16, 18, 31, 33, 52, cp, 58, Other(%)

A total of 2181 qualified specimens were collected. The specimens included 251 ICC cases and 1930 CIN cases. HPV infection was not detected in 200 cases. Infection with one, two, three, four, five, six, and eight types of HPV was detected in 1372 cases, 454 cases, 119 cases, 29 cases, 5 cases, 1 case, and 1 case, respectively. One 50-year-old patient with a CIN2 pathological diagnosis tested positive for HPV types 52, 39, 45, 51, 35, 53, 66, and CP8304.The distribution of HPV infection with a single type and multiple types in the study population from the Yangtze River Delta region is shown in Table 1 and Fig. 1.

**Table 1:** Distribution of HPV infection with a single type and multiple types in women from the Yangtze River Delta region

Types	CIN1		CIN2		CIN3		ICC		Total	
	n	(%)	n	(%)	n	(%)	n	(%)	n	(%)
Total	751		425		753		252		2181	
Total HPV (+)	666	(88.68)	392	(92.24)	701	(93.09)	222	(88.10)	1981	(90.83)
**16**	117	(17.57)	135	(34.44)	411	(58.63)	146	65.77)	809	(40.84)
**58**	167	(25.08)	95	(24.23)	129	(18.40)	20	(9.01)	411	(20.75)
**52**	184	(27.63)	75	(19.13)	93	(13.27)	19	(8.56)	371	(18.73)
**33**	59	(8.86)	47	(11.99)	104	(14.84)	15	(6.76)	225	(11.36)
**18**	41	(6.16)	20	(5.10)	32	(4.56)	27	(12.16)	120	(6.06)
**31**	38	(5.71)	29	(7.40)	54	(7.70)	9	(4.05)	130	(6.56)
**53**	38	(5.71)	25	(6.38)	37	(5.28)	12	(5.41)	112	(5.65)
**68**	34	(5.11)	16	(4.08)	27	(3.85)	10	(4.50)	87	(4.39)
**39**	32	(4.80)	11	(2.81)	12	(1.71)	7	(3.15)	62	(3.13)
**51**	31	(4.65)	11	(2.81)	6	(0.86)	2	(0.90)	50	(2.52)
**66**	30	(4.50)	8	(2.04)	10	(1.43)	1	(0.45)	49	(2.47)
**56**	30	(4.50)	4	(1.02)	7	(1.00)	1	(0.45)	42	(2.12)
**35**	10	(1.50)	8	(2.04)	5	(0.71)	1	(0.45)	24	(1.21)
CP	49	(7.36)	25	(6.38)	28	(3.99)	10	(4.50)	112	(5.65)
11	15	(2.25)	5	(1.28)	9	(1.28)	4	(1.80)	33	(1.67)
6	7	(1.05)	9	(2.30)	5	(0.71)	1	(0.45)	22	(1.11)
42	7	(1.05)	2	(0.51)	5	(0.71)	3	(1.35)	17	(0.86)

Note: Upright letters indicate HR-HPV’ italics indicate LR-HPV. HPV types with the frequency less than 1% are not shown

The proportion of HPV16 type in ICC is significantly different from that of other high-risk type HPV, P<0.01 (HPV58, χ^2^=173.40; HPV52, χ^2^=176.60; HPV33, χ^23^=189.83; HPV18, χ^2^=152.27; HPV31, χ^2^=211.23; HPV53, χ^2^=200.26; HPV68, χ^2^=207.55; HPV39, χ^2^=218.76; HPV51, χ^2^=238.59; HPV66, χ^2^=242.74; HPV56, χ^2^=242.74; HPV35, χ^2^=242.74)

The proportion of HPV18 type in ICC is significantly different from that of other high-risk type HPV, p<0.01 (HPV16, χ^2^=152.27; HPV31, χ^2^=11.13,; HPV53, χ^2^=7.16; HPV68, χ^2^=9.68; HPV39, χ^2^=14.47;HPV51, χ^2^=26.17; HPV66, χ^2^=29.25; HPV56, χ^2^=29.25;HPV35, χ^2^=29.25), except HPV58,52 and 33 type (HPV58, χ^2^=1.32, P>0.05, HPV52, χ^2^=1.76, p>0.05; HPV33, χ^2^=4.29, 0.01<p<0.05)

The proportion of HPV58 type in ICC is significantly different from that of other high-risk type HPV, P<0.01 (HPV16, χ^2^=173.40; HPV39, χ^2^=7.58; HPV51, χ^2^=17.60; HPV66, χ^2^=20.49; HPV56, χ^2^=20.49; HPV35, χ^2^=20.49), except HPV52,33,18,53 type (HPV52, χ^2^=0.03; HPV33, χ^2^=0.88; HPV18, χ^2^=1.32; HPV53, χ^2^=2.44), p>0.05 and HPV31, 68 type (HPV31, χ^2^=5.08; HPV68, χ^2^=4.07), 0.01<p<0.05

The proportion of HPV16 type in CIN3 is significantly different from that of other high-risk type HPV, P<0.01 (HPV58, χ^2^=257.32;HPV52, χ^2^=336.43; HPV33, χ^23^=310.65; HPV18, χ^2^=509.30; HPV31, χ^2^=440.58; HPV53, χ^2^=492.83; HPV68, χ^2^=525.97; HPV39, χ^2^=579.00; HPV51, χ^2^=601.28; HPV66, χ^2^=586.26; HPV56, χ^2^=597.56; HPV35, χ^2^=605.29)

The proportion of HPV58 type in CIN3 is significantly different from that of other high-risk type HPV, P<0.01 (HPV16, χ^2^=257.32; HPV52, χ^2^=7.43; HPV18, χ^2^=70.97; HPV31, χ^2^=37.99; HPV53, χ^2^=62.09; HPV68, χ^2^=80.61; HPV39, χ^2^=115.96; HPV51, χ^2^=133.10; HPV66, χ^2^=121.39; HPV56, χ^2^=130.14; HPV35, χ^2^=136.33), except HPV33 (HPV33, χ^2^=3.44), p>0.05

The proportion of HPV52 type in CIN3 is significantly different from that of other high-risk type HPV, P>0.01 (HPV16, χ^2^=336.43; HPV58, χ^2^=7.43; HPV18, χ^2^=35.17; HPV31, χ^2^=12.45; HPV53, χ^2^=28.56; HPV68, χ^2^=42.68; HPV39, χ^2^=72.61; HPV51, χ^2^=88.31; HPV66, χ^2^=77.51; HPV56, χ^2^=85.55; HPV35, χ^2^=91.36), except HPV33 (HPV33, χ^2^=0.77), p>0.05

The proportion of HPV16 type in CIN2 is significantly different from that of other high-risk type HPV, P<0.01 (HPV58, χ^2^=10.69; HPV52, χ^2^=25.40; HPV33, χ^2^=60.08; HPV18, χ^2^=115.33; HPV31, χ^2^=93.92; HPV53, χ^2^=103.00; HPV68, χ^2^=125.96; HPV39, χ^2^=140.27; HPV51, χ^2^=140.27; HPV66, χ^2^=149.58; HPV56, χ^2^=162.71; HPV35, χ^2^=149.58)

The proportion of HPV58 type in CIN2 is significantly different from that of other high-risk type HPV, P<0.01 (HPV16, χ^2^=10.69; HPV33, χ^2^=21.47; HPV18, χ^2^=62.14; HPV31, χ^2^=45.21; HPV53, χ^2^=52.23; HPV68, χ^2^=70.00; HPV39, χ^2^=83.39; HPV51, χ^2^=83.39; HPV66, χ^2^=91.71; HPV56, χ^2^=103.77; HPV35, χ^2^=91.71), except HPV52 (HPV52, χ^2^=3.26), p>0.05

The proportion of HPV52 type in CIN2 is significantly different from that of other high-risk type HPV, P<0.01 (HPV16, χ^2^=25.40; HPV33, χ^2^=8.25; HPV18, χ^2^=39.29; HPV31, χ^2^=25.41; HPV53, χ^2^=31.04; HPV68, χ^2^=46.92; HPV39, χ^2^=57.95; HPV51, χ^2^=57.95; HPV66, χ^2^=65.58; HPV56, χ^2^=76.93; HPV35, χ^2^=65.58), except HPV58 (HPV58, χ^2^=3.26), p>0.05

The proportion of HPV16 type in CIN1 is significantly different from that of other high-risk type HPV, P<0.01 (HPV58, χ^2^=12.62; HPV52, χ^2^=21.72; HPV33, χ^2^=24.84; HPV18, χ^2^=46.75; HPV31, χ^2^=51.35; HPV53, χ^2^=51.35; HPV68, χ^2^=57.98; HPV39, χ^2^=61.64; HPV51, χ^2^=63.47; HPV66, χ^2^=65.34; HPV56, χ^2^=65.34; HPV35, χ^2^=112.42)

The proportion of HPV58 type in CIN1 is significantly different from that of other high-risk type HPV, P<0.01 (HPV16, χ^2^=12.62; HPV33, χ^2^=70.11; HPV18, χ^2^=101.98; HPV31, χ^2^=108.17; HPV53, χ^2^=108.17; HPV68, χ^2^=116.84; HPV39, χ^2^=121.53; HPV51, χ^2^=123.84; HPV66, χ^2^=126.19; HPV56, χ^2^=126.19; HPV35, χ^2^=181.18), except HPV52 (HPV52, χ^2^=1.26),p>0.05

The proportion of HPV52 type in CIN1 is significantly different from that of other high-risk type HPV, P<0.01 (HPV16, χ^2^=21.72; HPV33, χ^2^=88.69; HPV18, χ^2^=123.28; HPV31, χ^2^=129.88; HPV53, χ^2^=129.88; HPV68, χ^2^=139.10; HPV39, χ^2^=144.06; HPV51, χ^2^=146.50; HPV66, χ^2^=148.98; HPV56, χ^2^=148.98; HPV35, χ^2^=206.04), except HPV58 (HPV58, χ^2^=1.26),p>0.05

The five most prevalent HPV types in ICC and its precursor patients from the Yangtze River Delta region were 16, 58, 52, 33, and 31. Of these, the most prevalent types were 52, 58, 16, 33, and CP8304 in CIN 1; 16, 58, 52, 33, and 31 in CIN 2; 16, 58, 33, 52, and 31 in CIN 3; and 16, 18, 58, 52, and 33 in ICC.

**Fig. 2:**
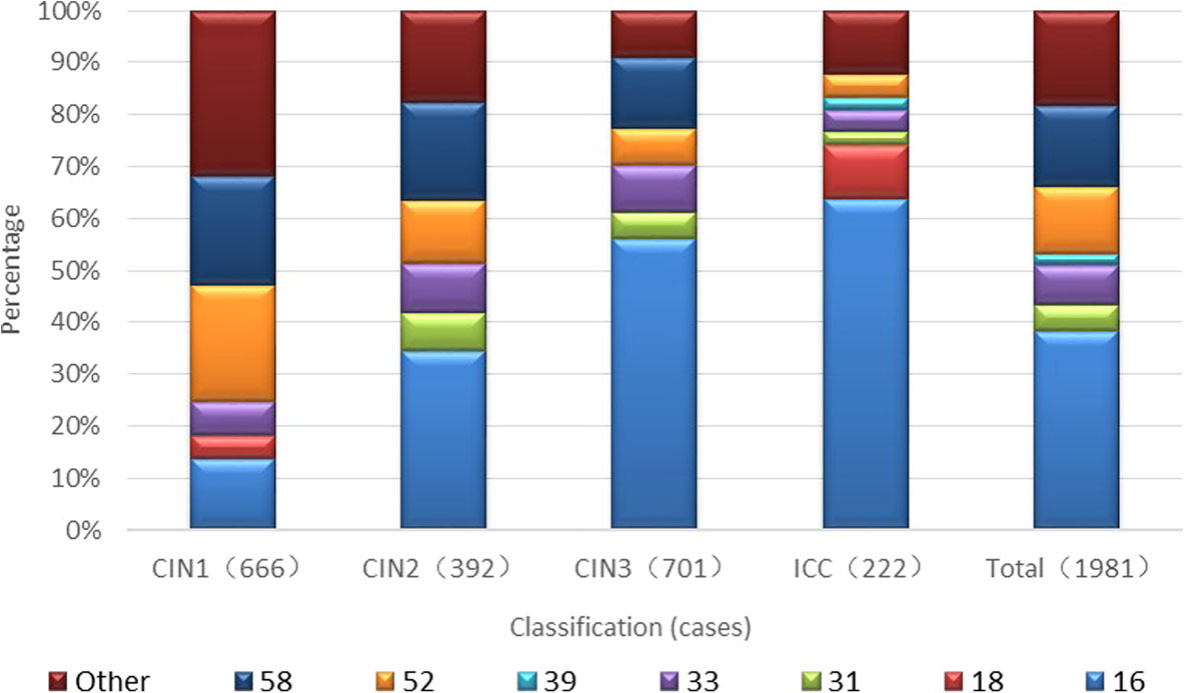
Distribution of infection with a single HPV type in women from the Yangtze River Delta region. (HPV)16, 18, 31, 33, 39, 52, 58, Other(%).

The distribution of infection with a single HPV type in the study population from the Yangtze River Delta region is shown in Fig. 2.

The five most prevalent HPV types involving a single infection were 16, 58, 52, 33, and 31 in ICC and CIN patients from the Yangtze River Delta region. Of these, the five most prevalent HPV types of single infection were 52, 58, 16, 33, and CP8304 in CIN 1; 16, 58, 52, 33, and 31 in CIN 2; 16, 58, 33, 52, and 31 in CIN 3; and 16, 18, 52, 33, and 31 in ICC.

The most prevalent HPV types were similar between single and multiple infections, suggesting that these types possess the strongest oncogenicity.

### Frequency of infection with a single or multiple HPV types in the Yangtze River Delta region

**Fig. 3:**
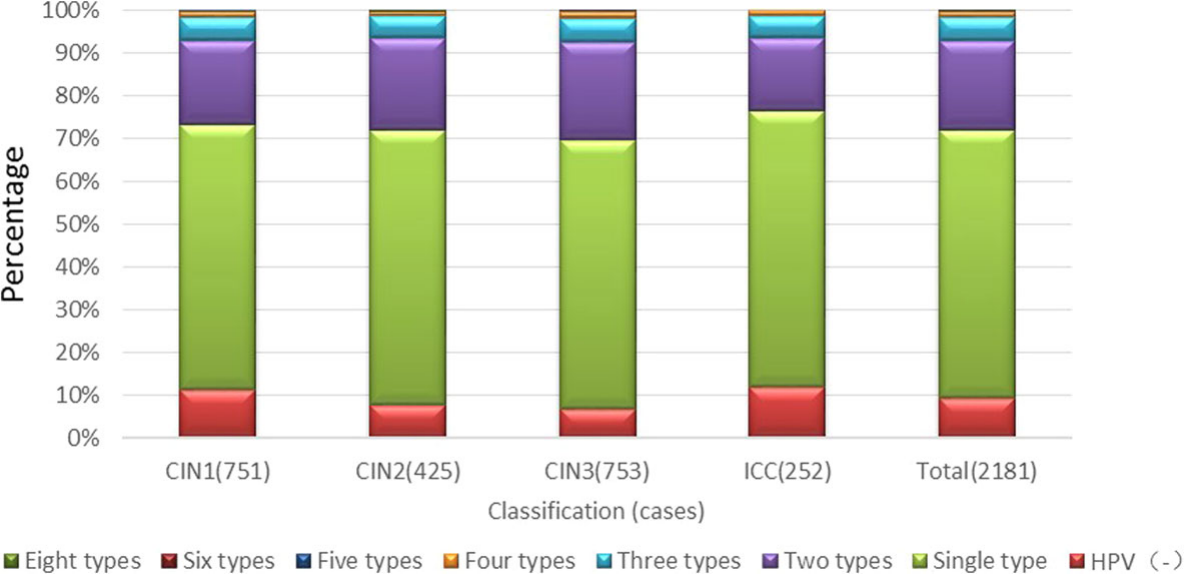
Frequency of multiple HPV types stratified according to the pathological results in the three study regions. HPV(−), (HPV)Single type, Two types, Three types, Four types, Five types, Six types, Eight types (%)

In all infected patients, the frequency of HPV infection with a single type was 62.9%, and with multiple types it was 38.1%. The most common number of multiple infections involved two different types, and the maximum number of types was eight. There were no differences in the frequencies of multiple types amongst different cervical lesions, suggesting that increased numbers of HPV types do not elevate the risk for high-grade CIN in ICC development, as shown in Fig. 3.

### Frequency of multiple HPV types in the three regions (Table 2)

**Table 2:** Frequency of multiple HPV types in women from the three study regions

Area	TTotal	Not detected (HPV-)		Types (%) of HPV(+)													
		n	(%)	Single type		Two types		Three types		Four types		Five types		Six types		Eight types	
Zhejiang	941	94	(9.99)	603	(64.08)	183	(19.45)	50	(5.31)	8	(0.85)	2	(0.21)	1	(0.11)	0	(0.00)
Jiangsu	784	69	(8.80)	473	(60.33)	170	(21.68)	49	(6.25)	19	(2.42)	3	(0.38)	0	(0.00)	1	(0.13)
Shanghai	456	37	(8.11)	296	(64.91)	102	(22.37)	19	(4.17)	2	(0.44)	0	(0.00)	0	(0.00)	0	(0.00)
Total	2181	200	(9.17)	1372	(62.91)	454	(20.82)	119	(5.46)	29	(1.33)	5	(0.23)	1	(0.05)	1	(0.05)

The incidence of multiple-type HPV infections in Jiangsu Province was significantly higher (*p* < 0.05) than in either Zhejiang Province or Shanghai

### Regional distribution of disease and age in the Yangtze River Delta region (Table 3)

**Table 3:** Age distribution of patients with cervical lesions in the Yangtze River Delta region

Age (years)	Total		CIN1		CIN2		CIN3		ICC	
	n	(%)	n	(%)	n	(%)	n	(%)	n	(%)
≤24	43	(1.97)	21	(2.80)	12	(2.83)	9	(1.19)	1	(0.40)
25–29	201	(9.22)	95	(12.65)	40	(9.43)	60	(7.95)	6	(2.39)
30–34	338	(15.50)	128	(17.04)	73	(17.22)	116	(15.36)	21	(8.37)
35–39	395	(18.11)	134	(17.84)	81	(19.10)	144	(19.07)	36	(14.34)
40–44	404	(18.52)	134	(17.84)	67	(15.80)	150	(19.87)	53	(21.12)
45–49	306	(14.03)	99	(13.18)	60	(14.15)	109	(14.44)	38	(15.14)
50–54	191	(8.76)	45	(5.99)	44	(10.38)	61	(8.08)	41	(16.33)
55–59	100	(4.59)	29	(3.86)	21	(4.95)	33	(4.37)	17	(6.77)
≥60	203	(9.31)	66	(8.79))	26	(6.13)	73	(9.67)	38	(15.14)
Total	2181	(100)	751	(100)	424	(100)	755	(100)	251	(100)

In the Yangtze River Delta region, over 10% of cervical cancer patients are under 35 years of age, and over 25% of patients with CIN and cervical cancer are under 35 years of age

### Age distribution of CIN and ICC in Zhejiang Province (Table 4)

**Table 4:** Age distribution of CIN and ICC in Zhejiang Province

Age (years)	Total		CIN1		CIN2		CIN3		ICC	
	n	(%)	n	(%)	n	(%)	n	(%)	n	(%)
≤24	17	(1.81)	14	(3.29)	2	(3.28)	0	(0.00)	1	(0.65)
25–29	89	(9.46)	61	(14.35)	6	(9.84)	21	(6.98)	1	(0.65)
30–34	140	(14.88)	80	(18.82)	6	(9.84)	42	(13.95)	12	(7.79)
35–39	135	(14.35)	63	(14.82)	3	(4.92)	50	(16.61)	19	(12.34)
40–44	166	(17.64)	75	(17.65)	6	(9.84)	48	(15.95)	37	(24.03)
45–49	143	(15.20)	58	(13.65)	10	(16.39)	49	(16.28)	26	(16.88)
50–54	71	(7.55)	10	(2.35)	9	(14.75)	27	(8.97)	25	(16.23)
55–59	47	(4.99)	15	(3.53)	10	(16.39)	16	(5.32)	6	(3.90)
≥60	133	(14.13)	49	(11.53)	9	(14.75)	48	(15.95)	27	(17.53)
Total	941	(100)	425	(100)	61	(100)	301	(100)	154	(100)

In Zhejiang Province, the incidence of cervical cancer in patients under 35 years old was 9.09%. The incidence of ICC and CIN in patients under 35 years old was 26.05%

### Age distribution of CIN and ICC patients in Jiangsu Province (Table 5)

**Table 5:** Age distribution of CIN and ICC patients in Jiangsu Province

Age (years)	Total		CIN1		CIN2		CIN3		ICC	
	n	(%)	n	(%)	n	(%)	n	(%)	n	(%)
≤24	19	(2.42)	2	(1.23)	9	(3.70)	8	(2.61)	0	(0.00)
25–29	69	(8.80)	15	(9.26)	20	(8.23)	29	(9.45)	5	(6.94)
30–34	148	(18.88)	27	(16.67)	54	(22.22)	59	(19.22)	8	(11.11)
35–39	167	(21.30)	36	(22.22)	53	(21.81)	66	(21.50)	12	(16.67)
40–44	145	(18.49)	32	(19.75)	39	(16.05)	65	(21.17)	9	(12.50)
45–49	91	(11.61)	15	(9.26)	28	(11.52)	38	(12.38)	10	(13.89)
50–54	66	(8.42)	15	(9.26)	19	(7.82)	21	(6.84)	11	(15.28)
55–59	36	(4.59)	9	(5.56)	10	(4.12)	8	(2.61)	9	(12.50)
≥60	43	(5.48)	11	(6.79)	11	(4.53)	13	(4.23)	8	(11.11)
Total	784	(100)	162	(100)	243	(100)	307	(100)	72	(100)

In Jiangsu Province, the incidence of cervical cancer in patients less than 35 years old was 18.05%. The incidence of ICC and CIN in patients less than 35 years old was 30.1%

### Age distribution of CIN and ICC patients in Shanghai (Table 6)

**Table 6:** Age distribution of CIN and ICC patients in Shanghai

Age (years)	Total		CIN1		CIN2		CIN3		ICC	
	n	(%)	n	(%)	n	(%)	n	(%)	n	(%)
≤24	7	(1.54)	5	(3.05)	1	(0.83)	1	(0.68)	0	(0.00)
25–29	43	(9.43)	19	(11.59)	14	(11.67)	10	(6.80)	0	(0.00)
30–34	50	(10.96)	21	(12.80)	13	(10.83)	15	(10.20)	1	(4.00)
35–39	93	(20.39)	35	(21.34)	25	(20.83)	28	(19.05)	5	(20.00)
40–44	93	(20.39)	27	(16.46)	22	(18.33)	37	(25.17)	7	(28.00)
45–49	72	(15.79)	26	(15.85)	22	(18.33)	22	(14.97)	2	(8.00)
50–54	54	(11.84)	20	(12.20)	16	(13.33)	13	(8.84)	5	(20.00)
55–59	18	(3.95)	5	(3.05)	1	(0.83)	9	(6.12)	3	(12.00)
≥60	26	(5.70)	6	(3.66)	6	(5.00)	12	(8.16)	2	(8.00)
Total	456	(100)	164	(100)	120	(100)	147	(100)	25	(100)

In Shanghai, the incidence of cervical cancer in patients less than 35 years old was 4%. The incidence of ICC and CIN in patients less than 35 years old was 21.93%

### Age-stratified HPV distribution in the study population from the Yangtze River Delta region

HPV types with the frequency less than 1% are not shown.

### Region-stratified HPV distribution in the study population from the Yangtze River Delta region

**Fig. 4:**
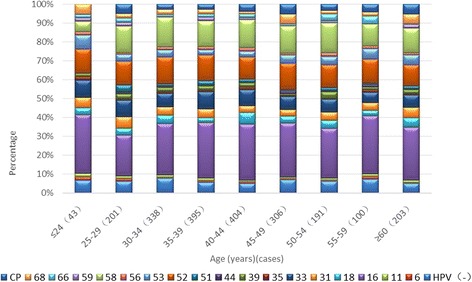
Age-stratified HPV distribution in CIN and ICC patients in the Delta Region (including multiple-type infection). HPV(−), (HPV)6, 11, 16, 18, 31, 33, 35, 39, 44, 51, 52, 53, 56, 58, 59, 66, 68, cp (%)

Age-stratified HPV distribution in the study population from the Yangtze River Delta region Fig 4. Age-stratified HPV distribution in CIN and ICC patients in the Delta Region (including multiple-type infection) HPV types with the frequency less than 1% are not shown.

HPV types with a frequency less than 1% are not shown.

HPV types with the frequency less than 1% are not shown.

**Fig. 5:**
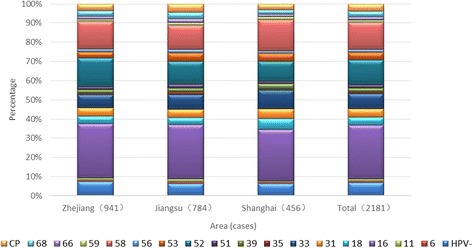
Comparison of HPV distributions in the three study regions (including multiple-type infections). HPV(−), (HPV)6, 11, 16, 18, 31, 33, 35, 39, 44, 51, 52, 53, 56, 58, 59, 66, 68, cp (%)

There was no significant difference in HPV typing in Jiangsu, Zhejiang and Shanghai (Fig. 5). HPV types with the frequency less than 1% are not shown.

### Regional distribution of disease and age in the Yangtze River Delta region, Zhejiang Province, Jiangsu Province and shanghai

**Fig. 6:**
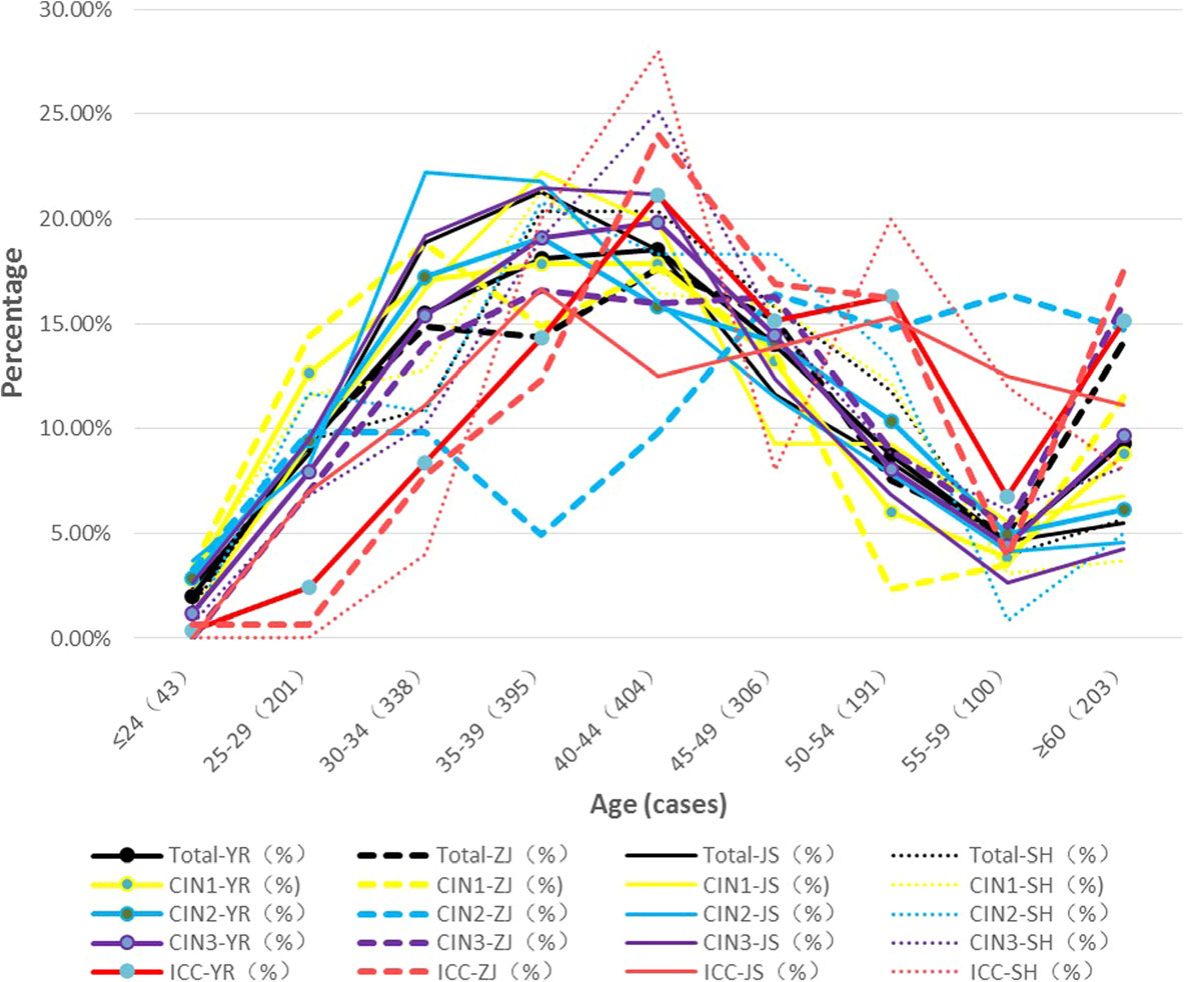
Statistical age (years) distribution of cervical lesions in the Yangtze River Delta region,Zhejiang Province, Jiangsu Province and Shanghai. ICC, CIN3, CIN2, CIN1, Total (%)

In the Yangtze River Delta region, over 10% of cervical cancer patients are under 35 years of age, and over 25% of patients with CIN and cervical cancer are under 35 years of age. The prevalence of CIN1 in the Delta region reached a peak in patients between 30 and 40 years of age, which is approximately 10 years earlier than the peak for other high-grade lesions. The prevalence of CIN3 and ICC peaked simultaneously and later than the peak for CIN2. ICC presented with two peaks at 40–44 and 50–54 years of age, as shown in Fig. 6. The Yangtze River Delta region = YR,Zhejiang Province = ZJ,Jiangsu Province = JS,Shanghai = SH,Of = −.For example (e.g.): Total of the Yangtze River Delta region(%) = Total-YR,CIN1 of Zhejiang Province = CIN1-ZJ.In Zhejiang Province, the incidence of cervical cancer in patients under 35 years old was 9.09%. The incidence of ICC and CIN in patients under 35 years old was 26.05%.The age distributions of patients with CIN1, CIN2, and ICC in Zhejiang Province all showed two peaks. The first peaks for CIN1 and CIN2 were 10 years earlier than that for ICC, and the first peak for CIN3 was 5 years earlier than that for ICC.In Jiangsu Province, the incidence of cervical cancer in patients less than 35 years old was 18.05%. The incidence of ICC and CIN in patients less than 35 years old was 30.1%.The age distribution of patients with ICC showed two peaks in Jiangsu Province. The first peak of Jiangsu Province for ICC was 5 years earlier than those of Zhejiang Province and Shanghai.In Shanghai, the incidence of cervical cancer in patients less than 35 years old was 4%. The incidence of ICC and CIN in patients less than 35 years old was 21.93%.The age distribution of patients with ICC showed two peaks in Shanghai. The prevalence of cervical lesions in patients less than 35 years old in Jiangsu Province was significantly higher (χ2 = 9.75, p < 0.01) than that in Shanghai. The age distribution of patients with ICC also showed two peaks in Shanghai, whereas those of CIN1 and CIN2 were 5 years earlier than that of ICC.

The Yangtze River Delta region = YR,Zhejiang Province = ZJ,Jiangsu Province = JS,Shanghai = SH,Of = −.For example (e.g.): Total of the Yangtze River Delta region(%) = Total-YR,CIN1 of Zhejiang Province = CIN1-ZJ.

In Zhejiang Province, the incidence of cervical cancer in patients under 35 years old was 9.09%. The incidence of ICC and CIN in patients under 35 years old was 26.05%.

The age distributions of patients with CIN1, CIN2, and ICC in Zhejiang Province all showed two peaks. The first peaks for CIN1 and CIN2 were 10 years earlier than that for ICC, and the first peak for CIN3 was 5 years earlier than that for ICC.

In Jiangsu Province, the incidence of cervical cancer in patients less than 35 years old was 18.05%. The incidence of ICC and CIN in patients less than 35 years old was 30.1%.

The age distribution of patients with ICC showed two peaks in Jiangsu Province. The first peak of Jiangsu Province for ICC was 5 years earlier than those of Zhejiang Province and Shanghai.

In Shanghai, the incidence of cervical cancer in patients less than 35 years old was 4%. The incidence of ICC and CIN in patients less than 35 years old was 21.93%.

The age distribution of patients with ICC showed two peaks in Shanghai. The prevalence of cervical lesions in patients less than 35 years old in Jiangsu Province was significantly higher (χ^2^ = 9.75, *p* < 0.01) than that in Shanghai. The age distribution of patients with ICC also showed two peaks in Shanghai, whereas those of CIN1 and CIN2 were 5 years earlier than that of ICC.

## Discussion

### Distribution of HPV types in the Yangtze River Delta region in CIN and ICC women

An excess of 120 HPV types have been identified to date, and over 40 types are associated with genital diseases. HPV types are classified as either HR-HPV or LR-HPV based on their carcinogenic potential. HR-HPV is associated with CIN2/3 and ICC and includes types 16, 18, 31, 33, 35, 45, 51, 52, 53, 56, 58, 59, 66, and 68. LR-HPV is associated with genital warts and LSIL and includes types 6, 11, 42, 43, and 44 [10].

Our present study identified that the most common HR-HPV types in CIN and ICC patients from the Delta region are HPV-16, 58, 52, 33, and 31. The distribution of infected HPV types shows substantial differences amongst healthy women, CIN and ICC patients. In a study in Zhejiang Province, Ye et al. reported an HPV prevalence of 13.3% using a cervical pap test. In the same population, the prevalence of HR-HPV was 10.2% [14]. The prevalence of individual HPV types was 3.1%, 2.5%, 2.1%, 1%, and 0.9% for types 52, 16, 58, 68, and 81, respectively. HPV 16 and 18 accounted for 3.1% of all samples, whereas other types accounted for 3.5% (*n* = 174). In a study from Nanjing, Jiangsu Province, Hong et al. reported overall prevalence of HPV DNA amongst pregnant women of 13.4% (422/3139). The most frequently detected HPV genotypes were HPV-16 (29.6%, 125/422), − 18 (14.7%, 62/422), and − 58 (14.2%, 60/422) [13]. The paper with the investigation of Ye and Hong in healthy women and cervical cancer HPV type is not exactly the same.

The distribution of infected HPV types shows substantial regional differences amongst CIN and ICC patients. Yang et al. previously investigated 1715 cervical neoplasia outpatients and identified HPV infection in 57.1% of those patients. They also reported that 76.9% of cancer patients were HPV-positive whereas 98.1% of CIN3 patients were HPV-positive. The most common types of HPV infection in these patients were HPV-16, 58, 52, 33, and 31 [15]. Tang et al. found an HPV infection rate of 25.2% in patients with cervical disease, reflecting the trend of increased incidence in recent years. The most common HR-HPV types detected by Tang et al. were 16, 58, 33, 52, and 18, with the distribution of HPV infection showing population differences and indicating that patients infected with a single HPV type may be more vulnerable to carcinogenesis [16]. In a randomized study, Munoz et al. found that the distribution of HPV types in the general population was similar to that in cervical cancer patients, with the most common being 16, 18, 45, 31, 58, 33, and 35 [10].

A meta-analysis of the HPV type distribution identified a total of 14,595 and 7094 cases of ICC and HSIL, respectively [9]. Worldwide, HPV16 was the most common type in ICC, followed by HPV18. In Asia, they were 16, 18, 58, 33, 52, 45, 31, and 35. In Asia, the HPV types in HSIL were 16, 58, 52, 18, 51, 33, 31, and 56. In ICC, HPV16 was the most common, with HPV18 the second most common type in all continents. The combined HPV16/18 prevalence amongst ICC cases was 65–70%. The next most common HPV types were the same in each continent, namely, HPV31, 33, 35, 45, 52, and 58; however, their relative importance differed somewhat by region. HPV18 was significantly more prevalent in adeno/adenosquamous carcinoma than in squamous cell carcinoma, with the reverse being true for HPV16, 31, 33, 52 and 58. Amongst HSIL cases, the HPV16/18 prevalence was 52%. However, HPV16, 18, and 45 were significantly under-represented, and other high-risk HPV types were significantly over-represented in HSIL compared with those in ICC, suggesting differences in type-specific risks for progression.

### HPV vaccination has good prospects in Yangtze River Delta

In the Yangtze River Delta region, the HPV type distribution of women aged over 35 years was similar to that of the HSIL type in women with CIN2 and CIN3 [17]. Furthermore, the distribution of the 5 sites was similar to that of HPV type. Moreover, the HPV type distribution in women aged 25–29 years was similar to the ratio of the first 5 columns of the HPV type distribution in LSIL (CIN1) women.

We hope that this cohort study provides information to support vaccination before the peak age of HPV infection. The best time to vaccinate women against HPV is before engaging in sexual activities, or at minimum prior to 13–24 years of age.

The HPV distribution frequency is correlated to some extent with pathogenicity. For example, women with persistent infection were more likely to have a higher pathogenicity. Our current findings suggested that in groups aged under 30 and over 40 years old, the highest frequency distribution was consistent with high pathogenicity and was observed in HPV types 16, 18, 58, 52, 33, and 31.

According to the most prevalent genotypes in our area, vaccination is a strong tool in the prevention of cervical cancer (especially for 16, 18 52, and 58, genotypes, from 9-valent HPV vaccine), and new vaccines are necessary to cover some genotypes that are missing from current formula.

In CIN patients, the most prevalent HPV type was HPV-52, and infections with multiple types were identified in 10% of individuals in the Yangtze River Delta Region. Jiangsu Province was found to have the most multiple-type infections. Our findings differ from those of the Beijing University First Hospital, Sichuan University, and Munoz, which all suggest a regional difference in HPV distribution between CIN and ICC. Our study indicates that HPV types 58 and 52, in addition to types 16 and 18, play important roles in the development of cervical cancer in the Yangtze River Delta Region.

There was no significant difference in the number of sexual partners or sexual behaviour of women in different areas of the Yangtze River Delta region. With respect to the economy, the GDP of Jiangsu Province was greater than that of Shanghai, which was greater than that of Zhejiang Province, whereas the national per capita income was the highest in Shanghai, followed by Zhejiang Province and Jiangsu Province. The per capita income is relatively low in Jiangsu Province; to increase this, the region has observed a large influx of personnel, resulting in an increased likelihood of HPV infection. Moreover, investigations into cervical cancer screening suggest an overall lack of health care knowledge and indicate that women do not pay attention to screening and early diagnosis and treatment of disease. This appears to be most serious in Jiangsu Province, followed by Zhejiang Province and Shanghai Province. Comparing the differences in the most common HPV types together with aspects of infection in the three different regions, we can conclude that the Jiangsu area has a higher proportion of composite infection. This likely reflects the rapid economic development of Jiangsu Province, the relatively low per capita income, the increased flow of personnel, the lack of health care knowledge, and the low importance placed on cervical cancer screening and early diagnosis and treatment of disease.

## Conclusions

HPV58 and HPV52, in addition to HPV16 and HPV18, may be involved in the occurrence of cervical cancer in the Yangtze River Delta area in China. Infection with multiple high-risk HPV types does not increase the risk for high-grade CIN in ICC development.

In the Yangtze River Delta Region, the peak age of the onset of cervical cancer was between 40 and 54 years, whereas that of CIN was between 30 and 39 years of age. On average, the peak age of onset of CIN was 5–10 years earlier than that of cervical cancer. The most common HPV types identified were 52, 58, 16, 33, and CP8304 in CIN1 patients; 16, 58, 52, 33, and 31 in CIN2 patients; 16, 58, 33, 52, and 31 in CIN3 patients; and 16, 18, 58, 52, and 33 in ICC patients. The age at which infection with HR-HPV most commonly occurs is the same as that in CIN and is earlier than the peak onset of cervical cancer. This is consistent with current epidemiological models of viral carcinogenesis.

According with the most prevalent genotypes in our area, vaccination is a strong tool in the prevention of cervical cancer, and new vaccines are necessary to cover some genotypes that are missing from the current formula.

## Abbreviations

CIN: Cervical intraepithelial neoplasia
HCll: Hybrid CaptureII
HPV: Human papillomavirus
HR-HPV: High risk human papilloma virus
HSIL: High-grade squamous intraepithelial lesion
ICC: Invasive cervical cancer
JS: Jiangsu Province
LSIL: Low-grade squamous intraepithelial lesion
SH: Shanghai
YR: The Yangtze River Delta region
ZJ: Zhejiang Province
